# Does surgical technique influence the burden of lung metastases in patients with pathologic long bone fractures?

**DOI:** 10.1186/s12891-022-05067-5

**Published:** 2022-01-31

**Authors:** Joseph K. Kendal, Bryan J. Heard, Annalise G. Abbott, Scott W. Moorman, Raghav Saini, Shannon K. T. Puloski, Michael J. Monument

**Affiliations:** 1 Section of Orthopaedic Surgery, Department of Surgery, University of Calgary, Calgary, Canada; 2 McCaig Institute for Bone and Joint Health, University of Calgary, Calgary, Canada; 3 Faculty of Medicine and Dentistry, University of Alberta, Edmonton, Canada; 4 Northern Ontario School of Medicine, Thunder Bay, Canada; 5 Foothills Medical Center, McCaig Tower, 3134 Hospital Drive N. W, Calgary, Alberta Canada

**Keywords:** Metastatic bone disease, Lung metastasis, Intramedullary nail, Pathologic fracture, Arthroplasty

## Abstract

**Background:**

The aims of this study are to (1) determine whether fixation of metastatic long bone fractures with an intramedullary nail (IMN) influences the incidence of lung metastasis in comparison to arthroplasty or ORIF (Arthro/ORIF); and (2) assess this relationship in primary tumor types; and (3) to assess survival implications of lung metastasis after surgery.

**Methods:**

Retrospective cohort study investigating 184 patients (107 IMN, and 77 Arthro/ORIF) surgically treated for metastatic long bone fractures. Patients were required to have a single surgically treated impending or established pathologic fracture of a long bone, pre-operative lung imaging (lung radiograph or computed tomography) and post-operative lung imaging within 6 months of surgery. Primary cancer types included were breast (*n* = 70), lung (*n* = 43), prostate (*n* = 34), renal cell (*n* = 37). Statistical analyses were conducted using two-tailed Fisher’s exact tests, and Kaplan-Meier survival analyses.

**Results:**

Patients treated with IMN and Arthro/ORIF developed new or progressive lung metastases following surgery at an incidence of 34 and 26%, respectively. Surgical method did not significantly influence lung metastasis (*p* = 0.33). Furthermore, an analysis of primary cancer subgroups did not yield any differences between IMN vs Arthro/ORIF. Median survival for the entire cohort was 11 months and 1-year overall survival was 42.7% (95% CI: 35.4–49.8). Regardless of fixation method, the presence of new or progressive lung metastatic disease at follow up imaging study was found to have a negative impact on patient survival (*p* < 0.001).

**Conclusions:**

In this study, development or progression of metastatic lung disease was not affected by long bone stabilization strategy. IM manipulation of metastatic long bone fractures therefore may not result in a clinically relevant increase in metastatic lung burden. The results of this study also suggest that lung metastasis within 6 months of surgery for metastatic long bone lesions is negatively associated with patient survival.

**Level of evidence:**

III, therapeutic study

## Background

As the efficacy of treatment options available to cancer patients has improved, so has the patient’s average life expectancy following a cancer diagnosis [1]. Metastatic bone disease (MBD) results in weakened, pathologic bone that is prone to fracture, with considerable implications to patient quality of life, mobility, and mortality [2, 3]. As such, metastatic long bone fractures have a significant health care burden and are associated with a poor prognosis. The humerus, femur, and tibia are the common targets of long bone metastasis, particularly from breast, thyroid, renal cell, lung and prostate primary cancers. The femur is the most common long bone affected by metastasis, followed by the humerus and tibia [4].

Surgical strategies for pathological long bone fracture fixation have been well studied. Surgical management of metastatic long bone fractures, using various surgical techniques and implants (indicated by lesion size, matrix, location, and degree of bone destruction), is an effective, typically palliative intervention that can significantly improve patient quality of life, including pain and mobility [5, 6]. While striving for these goals, surgical decision making in this patient population must also include patient-oriented life expectancy, timely surgical care, health economics and implant longevity in the context of persistent bone pathology. For example, en bloc resection and reconstruction with a large tumour endoprosthesis may be indicated for patients with solitary metastatic disease, whereas intramedullary nail (IMN) fixation may be a preferred strategy in a patient with multiple sites of bony metastasis and a short life expectancy. The influence of orthopaedic techniques on patient outcomes and oncologic survival is also important, yet poorly understood. Further research in this latter priority is critical to ensure orthopaedic practices are equally evolving with advances in other therapeutic domains of cancer care.

Techniques for stabilization of metastatic long bone fractures include IMN fixation, reconstruction using either conventional long-stemmed arthroplasty implants (ex. hip hemiarthroplasty or tumor endoprosthetics), and osteosynthesis using open reduction and internal fixation (ORIF). As extensively documented in orthopaedic trauma literature, intramedullary manipulation of long bones results in intravasation of marrow contents and fat emboli, which are disseminated into pulmonary circulation [7]. As a corollary, a rise in circulating tumor cells after intramedullary manipulation could result in seeding of the lung parenchyma [8]. Of note, while arthroplasty techniques involve intramedullary manipulation with a femoral stem, the surgery typically involves a gross tumor debulking step and a large open vent at the proximal femur, which in theory would result in a much lower degree of pressurization, and less gross tumor bulk for extravasation compared to IMN techniques. The clinical relevance of the pressurization phenomenon is not well understood, and it is not clear if certain fracture fixation methods may pose a greater risk for pulmonary dissemination of tumor emboli than others. While IMN fixation of long bone pathological fractures is an effective and minimally invasive fixation strategy that can facilitate excellent post-surgical outcomes, the implications on the risk of iatrogenic spread of cancer to the lungs remains unknown. As there remains considerable clinical equipoise regarding the ideal fixation methods for metastatic long bone lesions, a greater understanding of how surgical technique influences oncologic outcome is warranted.

**Fig. 1:**
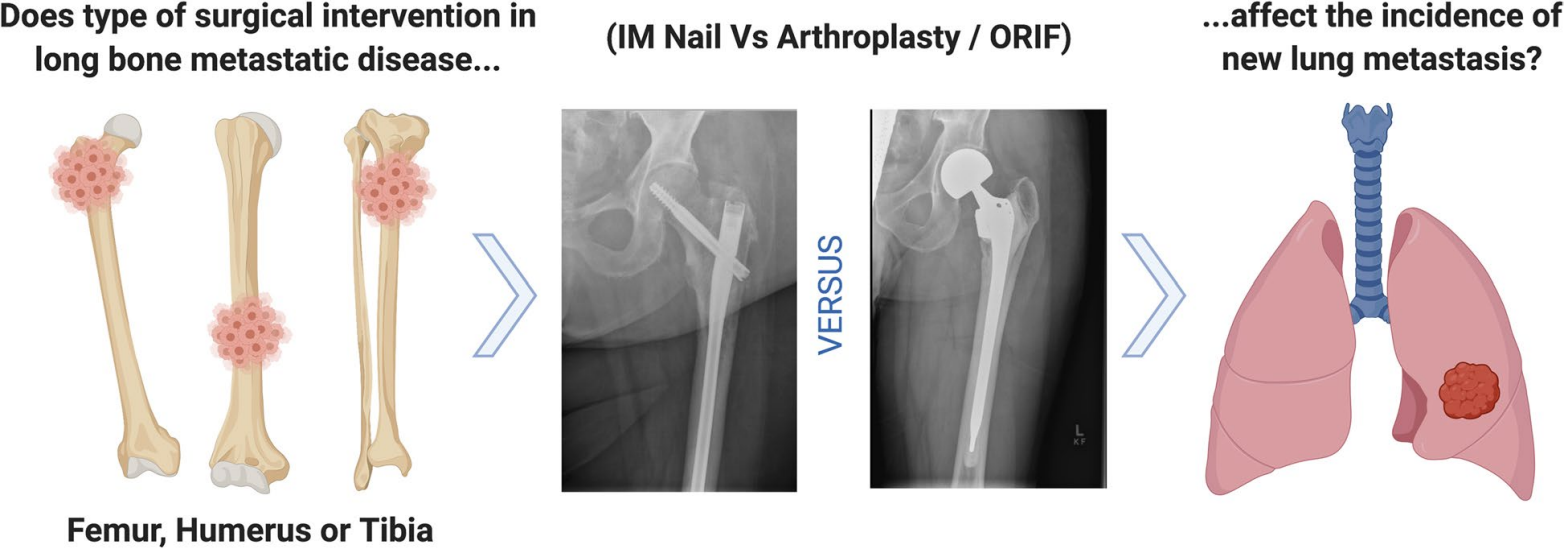
The primary research objective was to assess whether surgical technique influenced subsequent incidence of new or progressive lung metastases

The primary objective of this study was to determine if the surgical fixation of pathological fractures using IMN significantly increases the incidence of new metastatic disease to the lungs compared to arthroplasty and ORIF techniques (Arthro/ORIF) (Fig. 1). Secondary objectives include performing a sub-group analysis of breast, lung, prostate, and renal cell carcinoma primary tumors to describe incidence of new metastatic lung disease following IMN vs. Arthro/ORIF of pathological fracture within each of these primary cancer types, as well as the mortality associated with new or progressive lung metastases within 6 months of surgery.

## Methods

A retrospective cohort study was conducted under ethics approval from the University of Calgary Research Ethics Board, and the Health Research Ethics Board of Alberta Cancer Committee (REB16–2053 REN5).

### Patient selection

Patient selection and patent data acquisition was conducted through a provincial database query of urban zones in southern Alberta between April 2006 and January 2018. The database query included the following hospitals: Foothills Medical Center (Calgary, AB), Rockyview General Hospital (Calgary, AB), Peter Lougheed Hospital (Calgary, AB), South Health Campus (Calgary AB), Chinook Regional Hospital (Lethbridge, AB), Red Deer Regional Hospital (Red Deer, AB), St. Mary’s Hospital (Camrose, AB) and Medicine Hat Regional Hospital (Medicine Hat, AB). The cohort consisted of patients identified to have a fracture of the humerus, femur or tibia with a concurrent diagnosis of cancer, undergoing orthopedic surgical intervention.

### Inclusion criteria and classification

Included patients were required to have a single pathological fracture of a long bone secondary to metastatic bone disease. Patients must have also been treated surgically by an orthopedic surgeon, and had a primary cancer diagnosis of either breast, lung, prostate, or renal cell carcinoma. Patients must have had appropriate (as outlined below) pre- and post-operative chest imaging with either chest x-ray (CXR) or computed tomography (CT). Ongoing local or systemic cancer treatment, including radiotherapy, chemotherapy and immunotherapy were not used to stratify patient inclusion.

For patient classification as positive or negative for new lung metastasis the following conditions needed to be met: to be positive for disease progression in the lungs, chest imaging must be completed up to 3 months prior, and not more than 6 months post-operatively. This imaging must show either new disease, or increased disease burden in the lungs post-operatively, as quantified by the radiology report. To be negative for disease progression in the lungs, chest imaging must be completed any time following surgery that shows no disease more than 1 month, and not more than 2 years, post-operatively. Additionally, patients with pre-existing disease were considered negative if their lung disease burden was determined to be stable (or improved) at least 1 month post operatively by a radiologist.

### Exclusion criteria

Patients were excluded if they had more than one orthopedic surgical intervention or more than one pathologic fracture within 6 months. Patients were also excluded if they have incomplete chest imaging (as defined in inclusion criteria), or if the operative report and/or medical oncology reports confirming primary tumor diagnosis were unavailable.

### Data extraction

A data set of de-identified patient records was created for investigation using the following parameters: patient age, sex, primary cancer type, fracture type, orthopedic procedure, presence of pre- and post-operative imaging, presence of new lung metastasis, and date of death (if applicable). All patient data was stored on a secure, web-based database “Research Electronic Data Capture” (REDCap), developed in accordance with institutional regulations.

### Statistical analysis

All statistics were completed using two-tailed Fisher’s exact tests. Overall patient survival analysis was performed using the Kaplan-Meier method. The log-rank test was used to evaluate survival differences between groups. Statistical significance for all tests was accepted at *p* ≤ 0.05. All statistics were performed using GraphPad Prism version 5.0f for Mac OS X (GraphPad software, San Diego, CA, USA) and STATA (Statistics/Data Analysis 16.1, College Station, TX, USA).

## Results

### Patient population

**Fig. 2:**
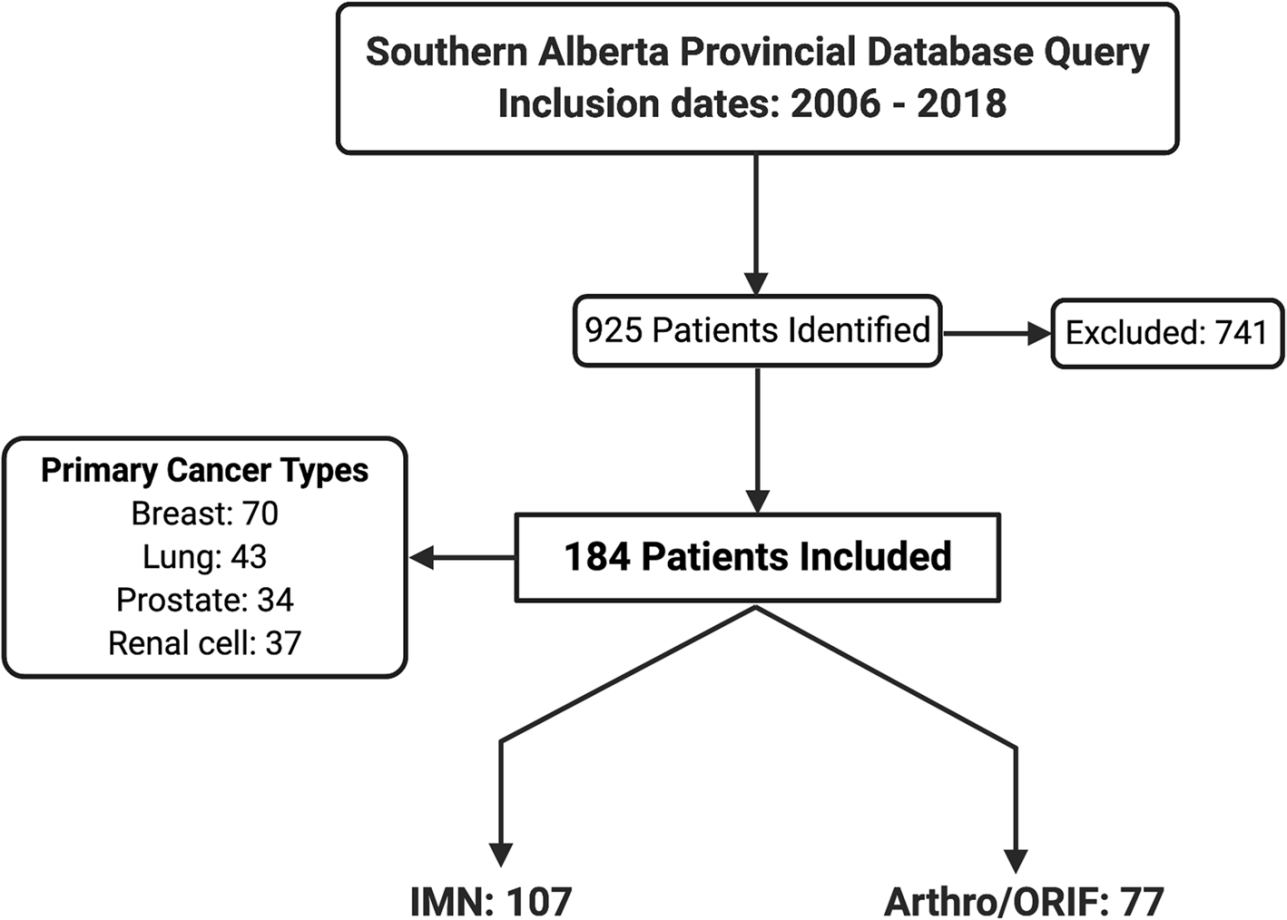
Details regarding the patient selection process are represented as a flow diagram

Of the 925 patients returned by our database search query, 184 met the inclusion/exclusion criteria (107 long IMN fixation, and 77 Arthro/ORIF) (Fig. 2). Of the Arthro/ORIF group, 20 patients underwent ORIF, and 57 patients underwent arthroplasty (3 patients had total joint arthroplasty, and the remaining 54 underwent hemiarthroplasty). The search strategy sensitivity was not able to exclude patients with concurrent cancer diagnoses undergoing orthopaedic trauma surgery, therefore a large number of patients retrieved required exclusion. See Table 1 for a summary of patient demographic data. The patients of the study were divided into stable (no new lung metastasis) and progressive (presence of new or progressive lung metastasis following pathological fracture fixation) cohorts for evaluation. Overall, the average age of patients was 65 years.

**Table 1:** Demographic characteristics of patients included in the study

Parameter	Total, N (%)	IMN, N (%)	ORIF/Arthro, N (%)
Patient Sex			
Female	108	63 (59)	45 (58)
Male	76	44 (41)	32 (42)
Age	65.3 (SD ±11.4)		
Primary Cancer			
Breast	70	37 (35)	33 (43)
Lung	43	30 (28)	13 (17)
Prostate	34	22 (20)	12 (15)
Renal	37	18 (17)	19 (25)
Long Bone Involved			
Humerus	24	9 (8)	15 (20)
Femur	156	97 (90)	59 (78)
Tibia	4	2 (2)	2 (2)
Location Within Bone			
Proximal Femur	107		
Femoral Diaphysis	39		
Distal Femur	10		
Proximal Humerus	9		
Humeral Diaphysis	12		
Distal Humerus	3		
Proximal Tibia	2		
Tibial Diaphysis	1		
Distal Tibia	1		
Pre-op Imaging Modality			
CXR	48	31 (29)	17 (22)
CT	133	75 (70)	58 (75)
None	3	1 (1)	2 (3)
Post-op Imaging Modality			
CXR	59	43 (40)	16 (21)
CT	125	64 (60)	61 (79)

### Incidence of new metastatic lung disease

**Fig. 3:**
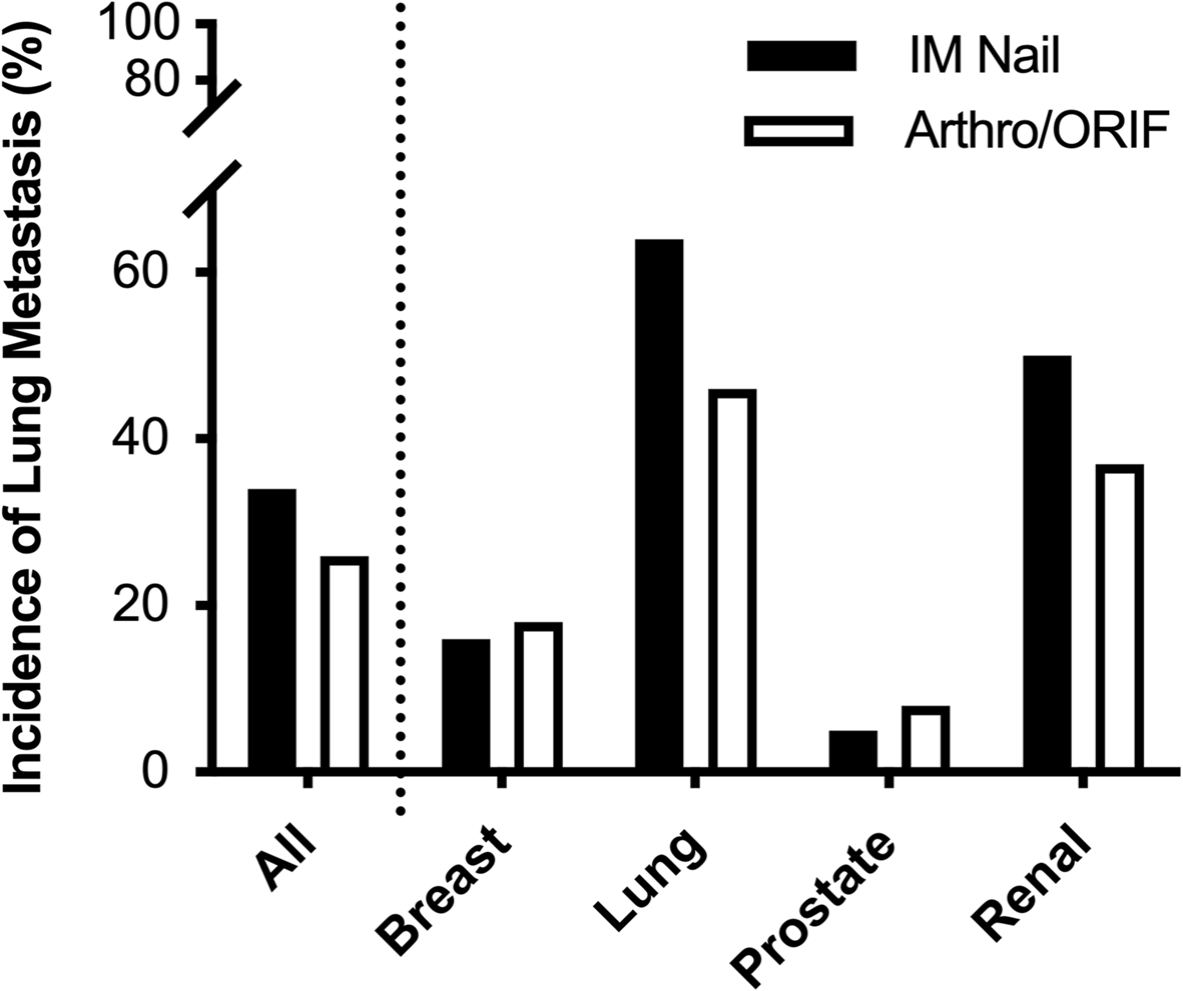
Summary data of the incidence of new or progressive lung metastasis demonstrated no difference in the incidence of lung metastasis for all primaries combined, or for breast, lung, prostate or renal primaries analyzed individually

As summarized in Table 2 and Fig. 3, in the entire study cohort, 34% (36/107) of patients with IMN fixation and 26% (20/77) of patients with Arthro/ORIF presented with new or progressive lung metastasis at follow up (*p* = 0.33). A sub-group analysis was conducted to investigate the incidence of new and progressive metastatic lung disease within breast, lung, prostate, and renal primary cancers. In breast cancer, 16% (6/37) of IMN fixations, compared to 18% (6/33) of Arthro/ORIF showed new or progressive lung metastatic disease (*p* = 1.00). For lung primaries, 67% (20/30) of IMN and 46% (6/13) of Arthro/ORIF patients showed new or progressive metastatic lung disease at follow up imaging study (*p* = 0.31). In patients with prostate cancer, 1 patient in each cohort developed new or progressive lung metastasis [5% (1/22) IMN vs. 8% (1/12) Arthro/ORIF, *p* = 1.00]. Finally, in patients with renal cell primaries, we found that 50% (9/18) of IMN, compared to 37% (7/19) of Arthro/ORIF, presented with new or progressive metastatic lung disease at the follow up imaging study (*p* = 0.52). A subgroup analysis of patients with lung cancer and renal cell cancer, two primaries with a high propensity for lung metastases, also did not show a significant association of surgical technique and new or progressive metastatic lung disease (*p* = 0.11). When specifically examining patients who were assessed with CT scans, there was no significant association between surgical technique and new or progressive metastatic lung disease (*p* = 0.35). In summary, no association between IMN and new metastatic lung disease compared to that of Arthro/ORIF was quantified the entire study cohort or in the subgroup analysis of breast, lung, prostate or renal cell cancer primary tumors (Table 2).

**Table 2:** Data summary of new or progressive lung metastasis detected post-operatively

Parameter	No Progression, N (%)	Progression, N (%)	*p*-Value
Mean Age (± SD)	66.2 (SD ±11.7)	63.2 (SD ±10.4)	
All Patients	128 (70)	56 (30)	
Surgical Technique			0.33
(All Primaries)			
Arthro/ORIF	57 (74)	20 (26)	
IMN	71 (66)	36 (34)	
Breast			1.00
Arthro/ORIF	27 (82)	6 (18)	
IMN	31 (84)	6 (16)	
Lung			0.31
Arthro/ORIF	7 (54)	6 (46)	
IMN	10 (33)	20 (67)	
Prostate			1.00
Arthro/ORIF	11 (92)	1 (8)	
IMN	21 (95)	1 (5)	
Renal			0.52
Arthro/ORIF	12 (63)	7 (37)	
IMN	9 (50)	9 (50)	

Statistical analysis was performed utilizing a two tailed Fischer’s exact test. *SD* standard deviation, *Arthro/ORIF* Arthroplasty and open reduction internal fixation cases combined, *IMN* Intramedullary nail

### Survival following surgical intervention of pathological fracture

**Fig. 4:**
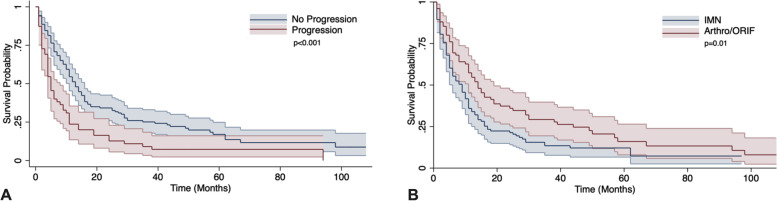
Survival analyses assessed the influence of progressive lung disease and surgical technique on mortality. **A** Kaplan Meier survival analysis was performed for patients who were identified to have no lung progression, versus those who had progression. **B** Kaplan Meier survival analysis was performed for patients undergoing either IMN or Arthro/ORIF. IMN = Intramedullary nail, Arthro/ORIF = arthroplasty and open reduction internal fixation cases combined. Shaded areas represent 95% confidence intervals

Median survival for the entire cohort was 11 months, and 1-year overall survival was 42.7% (95% CI: 35.4–49.8). Within the entire cohort, the 1-year overall survival was 23.6% (95% CI: 13.5–35.4) for those who experienced new or progressive lung metastasis within 6 months, compared to 52.2% (95% CI: 42.1–59.6) for those who did not experience new or progressive lung metastasis. On the Kaplan-Meier survival analysis, patients with no lung disease progression demonstrated improved survival in comparison to those who did experience new or progressive lung metastasis (Fig. 4A, *p* < 0.001). The 1-year overall survival of patients undergoing IMN fixation was 34.0% (95% CI: 25.0–43.1) compared to 54.7% (95% CI: 42.8–65.1) in those undergoing Arthro/ORIF reconstructions. On the Kaplan-Meier survival analysis, patients undergoing Arthro/ORIF demonstrated improved survival in comparison to those undergoing IMN fixation (Fig. 4B, *p* = 0.01).

## Discussion

There were three main findings of this study. First, when investigating the entire cohort, and secondly, a subgroup analysis of individual primaries (breast, lung, prostate, renal cell) there were no differences in the incidence of new or progressive lung metastasis following pathological fracture fixation with IMN compared to Arthro/ORIF. Third, when fixation methods were combined, new or progressive metastasis to the lungs following surgical intervention for pathologic fracture was associated with worsened survival outcomes. Furthermore, we also found IMN fixation was negatively associated with patient survival as compared to Arthro/ORIF.

The risk of iatrogenic systemic disease progression is a meaningful question to address. One-year mortality following pathological fracture fixation is historically significant, and unfortunately, we continue to appreciate a dismal prognosis for patients with metastatic bone disease requiring orthopaedic surgical intervention. Previous studies report 1 year overall survival between 30 and 40% when all primary cancers are considered [3]. The survival of patients within our study was comparable to these numbers (42.7, 95% CI: 35.4–49.8), however our analysis revealed a significant increase in risk of mortality in the presence of new lung metastatic disease following surgery compared to the stable group. This finding, however, may represent a surrogate marker for global systemic disease progression and physiologic deterioration. While we did not demonstrate a significant association between surgical technique and progression; it is important that further investigation be performed to assess other factors related to disease progression post-operatively. The iterative process of refining survival estimation algorithms (ex. PATHFx) using techniques such as machine learning requires accurate inputs of multiple factors predicting patient outcome [9, 10]. By furthering our understanding of surgical technical factors that are related to overall patient survival, we can more accurately counsel patients on treatment goals, and modify surgical techniques accordingly.

Interestingly, while surgical technique was not statistically associated with metastatic progression in the lung in this study, we did demonstrate that those who underwent IMN fixation experience a poorer prognosis than those undergoing reconstruction with either arthroplasty or ORIF techniques. There are many possible explanations for this observation as there are many factors that go into the surgical decision making for patients with MBD. The fracture pattern may dictate the fixation strategy, but also pre-operative survival estimates, the concomitant necessity for tumour debulking or resection, the number of metastases present (solitary vs. oligometastatic vs. polymetastatic disease) and surgeon and patient preferences, to name a few [11]. In particular, the bias towards IMN fixation when feasible in those with a very short life expectancy would confound the data and bias a worse prognosis in those treated with IMN [12]. Without standardizing pre-operative survival estimates, this confounding factor would be difficult to eliminate. Although the theoretical increase in circulating tumour cells with intramedullary manipulation did not result in an increase in new or progressive lung metastasis, perhaps there is another secondary sequela that is not accounted for in this study that negatively influences prognosis. Intramedullary manipulation may also lead to a relatively increased dissemination of tumour cells in impending fractures (vs. established fractures) due to increased intramedullary pressure changes compared to established fractures [13]. In general, surgical fixation of an impending pathological fracture bears a more favorable prognosis for the patient (and economic benefits to the health care system) than stabilizing an established pathological fracture [14, 15]. It is unclear, however, if the factor of impending vs. established fracture influences clinically relevant lung disease progression after IMN fixation. Future research should stratify cohorts according to impending vs. established fractures to help delineate this relationship.

### Limitations

There are limitations to this study. The presence of a pathologic fracture itself, regardless of the fixation type, has been theorized to lead to metastatic spread to the lungs [16]. This study included patients who had undergone surgery for both impending and established pathologic fractures. Future studies should compare the results of pathological fracture fixation vs impending pathological fracture fixation and the effect on systemic tumour burden. It is possible that the timing and sensitivity of the post-operative imaging studies did not capture a change in lung tumour burden. We used both CXR and CT to detect tumour burden; standardizing the assessment method would reduce the risk of bias associated with a potentially reduced sensitivity of detection with CXR. As we believe this clinical question is important and remains relevant, future prospective studies involving surgical MBD patients should include standardized evaluations of disease progression post-op and build on the data presented here. Furthermore, the sample sizes included, and particularly within each primary tumour type may not have been of sufficient size to detect a difference in new or progressive lung metastasis. In particular, renal cell cancer, which has an affinity for lung metastasis, [17] was associated with a 50% incidence of new or progressive lung metastasis in the IMN cohort, vs. 37% in the Arthro/ORIF cohort, with 37 patients included in this subgroup. Other potential confounding factors not analyzed include use of concomitant therapies such as chemotherapy, radiation therapy and immunotherapies, as well as the presence of visceral metastasis, and overall patient systemic health and functional status. This further emphasizes the need for using large, multi-center databases to study this heterogeneous disease process, to utilize appropriate power estimates and detect differences not only amongst the entire pooled cohort, but importantly amongst the individual primary cancer types [18].

## Conclusion

As the availability and efficacy of life preserving cancer treatments have improved, the incidence of pathological fractures resulting from metastatic bone disease has increased. In this study, we have described the incidence of lung metastasis after surgery for MBD pathologic fractures, and have provided evidence to suggest that fixation method may not be associated with an increased risk in new lung metastatic disease for specific primary cancer types (breast, prostate, renal, and lung). Furthermore, we have shown a significant association between patient mortality and the presence of new or progressive lung metastatic disease in the first six months following surgical management of pathological fractures. Further work is required to better inform surgical decision making in the selection of fixation methods to treat pathological fractures resulting from metastatic bone disease.

## Data Availability

Datasets used and analyzed contain patient information and are stored in a REDCap database. De-identified datasets available upon reasonable request to the corresponding author, Michael J Monument (mjmonume@ucalgary.ca).

## Abbreviations

Arthro: Arthroplasty
CT: Computed tomography
CXR: Chest x-ray
IMN: Intramedullary nail
MBD: Metastatic bone disease
ORIF: Open reduction and internal fixation
REDCap: Research electronic data capture
